# Activated *PTHLH* Coupling Feedback Phosphoinositide to G-Protein Receptor Signal-Induced Cell Adhesion Network in Human Hepatocellular Carcinoma by Systems-Theoretic Analysis

**DOI:** 10.1100/2012/428979

**Published:** 2012-09-10

**Authors:** Lin Wang, Juxiang Huang, Minghu Jiang, Hong Lin, Lianxiu Qi, Haizhen Diao

**Affiliations:** ^1^Biomedical Center, School of Electronic Engineering, Beijing University of Posts and Telecommunications, Beijing 100876, China; ^2^Lab of Computational Linguistics, School of Humanities and Social Sciences, Tsinghua University, Beijing 100084, China

## Abstract

Studies were done on analysis of biological processes in the same high expression (fold change ≥2) activated *PTHLH* feedback-mediated cell adhesion gene ontology (GO) network of human hepatocellular carcinoma (HCC) compared with the corresponding low expression activated GO network of no-tumor hepatitis/cirrhotic tissues (HBV or HCV infection). Activated *PTHLH* feedback-mediated cell adhesion network consisted of anaphase-promoting complex-dependent proteasomal ubiquitin-dependent protein catabolism, cell adhesion, cell differentiation, cell-cell signaling, G-protein-coupled receptor protein signaling pathway, intracellular transport, metabolism, phosphoinositide-mediated signaling, positive regulation of transcription, regulation of cyclin-dependent protein kinase activity, regulation of transcription, signal transduction, transcription, and transport in HCC. We proposed activated *PTHLH* coupling feedback phosphoinositide to G-protein receptor signal-induced cell adhesion network. Our hypothesis was verified by the different activated *PTHLH* feedback-mediated cell adhesion GO network of HCC compared with the corresponding inhibited GO network of no-tumor hepatitis/cirrhotic tissues, or the same compared with the corresponding inhibited GO network of HCC. Activated *PTHLH* coupling feedback phosphoinositide to G-protein receptor signal-induced cell adhesion network included *BUB1B, GNG10, PTHR2, GNAZ, RFC4, UBE2C, NRXN3, BAP1, PVRL2, TROAP,* and *VCAN* in HCC from GEO dataset using gene regulatory network inference method and our programming.

## 1. Introduction

*PTHLH* is one of our identified significant high expression (fold change ≥2) genes in human hepatocellular carcinoma (HCC) compared with low expression no-tumor hepatitis/cirrhotic tissues (HBV or HCV infection) from GEO data set GSE10140-10141 [1].

Study of* PTHLH *is presented in some papers, such as Mouse pthlh gene-specific expression profiles distinguish among functional allelic variants in transfected human cancer cells [2]; parathyroid hormone-like protein alternative messenger RNA splicing pathways in human cancer cell lines [3]; parathyroid hormone-like peptide in pancreatic endocrine carcinoma and adenocarcinoma associated with hypercalcemia [4]; parathyroid hormone and parathyroid hormone-like peptide bioactivity in situ biochemistry [5]; parathyroid hormone-like protein polypeptides immunological identification and distribution in normal and malignant tissues [6]; dysregulation of parathyroid hormone-like peptide expression and secretion in a keratinocyte model of tumor progression [7]; all major lung cancer cell types produce parathyroid hormone-like protein [8]; parathyroid hormone-like peptide in normal and neoplastic mesothelial cells [9]. Yet the high expression activated* PTHLH *feedback-mediated cell adhesion mechanism in HCC is not clear and remains to be elucidated.

In this study, biological processes and occurrence numbers of the same activated high expression (fold change ≥2) *PTHLH *feedback-mediated cell adhesion GO network in HCC were identified and computed compared with the corresponding low expression activated GO network of no-tumor hepatitis/cirrhotic tissues (HBV or HCV infection), the different compared with the corresponding inhibited *PTHLH* feedback-mediated cell adhesion GO network of no-tumor hepatitis/cirrhotic tissues, and the same compared with the corresponding inhibited GO network of HCC, respectively. Simultaneous occurrence of biological processes was identified between the same activated* PTHLH *feedback-mediated cell adhesion GO network of HCC (compared with the corresponding activated GO network of no-tumor hepatitis/cirrhotic tissues) and the different (compared with the corresponding inhibited *PTHLH *feedback-mediated cell adhesion GO network of no-tumor hepatitis/cirrhotic tissues), or the same (compared with the corresponding inhibited GO network of HCC) for putting forward hypothesis of activated* PTHLH *coupling feedback phosphoinositide to G-protein receptor signal-induced cell adhesion network. Activated *PTHLH* feedback-mediated cell adhesion molecular network and numbers in HCC were extracted and computed from the same activated* PTHLH* GO-molecular network of HCC compared with the corresponding activated GO-molecular network of no-tumor hepatitis/cirrhotic tissues. *PTHLH* coupling feedback phosphoinositide to G-protein receptor signal-induced cell adhesion molecular relationship in HCC was identified including different molecules but same GO term and same molecule but different GO terms from the same activated* PTHLH* GO-molecular network of HCC compared with the corresponding activated GO-molecular network of no-tumor hepatitis/cirrhotic tissues.

## 2. Materials and Methods

Microarray 6,144 genes were used for analyzing activated* PTHLH *feedback-mediated cell adhesion mechanism of HCC based on GEO data set GSE10140-10141 (http://www.ncbi.nlm.nih.gov/geo/query/acc.cgi?acc=GSE10140, http://www.ncbi.nlm.nih.gov/geo/query/acc.cgi?acc=GSE10141). The raw microarray data was preprocessed by log base 2. 

225 significant high expression (fold change ≥2) molecules in HCC compared with no-tumor hepatitis/cirrhotic tissues (HBV or HCV infection) were identified using significant analysis of microarrays (SAM) (http://www-stat.stanford.edu/~tibs/SAM/) [10]. We selected two classes paired and minimum fold change ≥2 under the false-discovery rate was 0%.

Activated* PTHLH *feedback-mediated cell adhesion mechanism of HCC was analyzed by using Molecule Annotation System, MAS (CapitalBio Corporation, Beijing, China; http://bioinfo.capitalbio.com/mas3/). The primary databases of MAS integrated various well-known biological resources, such as Gene Ontology (http://www.geneontology.org/), KEGG (http://www.genome.jp/kegg/), BioCarta (http://www.biocarta.com/), GenMapp (http://www.genmapp.org/), HPRD (http://www.hprd.org/), MINT (http://mint.bio.uniroma2.it/mint/Welcome.do), BIND (http://www.blueprint.org/), Intact (http://www.ebi.ac.uk/intact/), UniGene (http://www.ncbi.nlm.nih.gov/unigene), OMIM (http://www.ncbi.nlm.nih.gov/entrez/query.fcgi?db=OMIM), and disease (http://bioinfo.capitalbio.com/mas3/). 

Biological processes and occurrence numbers of the same activated high expression (fold change ≥2) *PTHLH *feedback-mediated cell adhesion GO network in HCC were identified and computed compared with the corresponding low expression activated GO network of no-tumor hepatitis/cirrhotic tissues (HBV or HCV infection), the different compared with the corresponding inhibited *PTHLH* feedback-mediated cell adhesion GO network of no-tumor hepatitis/cirrhotic tissues, and the same compared with the corresponding inhibited GO network of HCC by our programming, respectively.

Simultaneous occurrence of biological processes was identified between the same activated* PTHLH *feedback-mediated cell adhesion GO network of HCC (compared with the corresponding activated GO network of no-tumor hepatitis/cirrhotic tissues) and the different (compared with the corresponding inhibited *PTHLH *feedback-mediated cell adhesion GO network of no-tumor hepatitis/cirrhotic tissues), or the same (compared with the corresponding inhibited GO network of HCC) for putting forward hypothesis of activated* PTHLH* coupling feedback phosphoinositide to G-protein receptor signal-induced cell adhesion network by our programming, respectively.

Activated* PTHLH *feedback-mediated cell adhesion molecular network and numbers in HCC were extracted and computed from the same activated* PTHLH* GO-molecular network of HCC compared with the corresponding activated GO-molecular network of no-tumor hepatitis/cirrhotic tissues by our programming, respectively. 

At last, *PTHLH* coupling feedback phosphoinositide to G-protein receptor signal-induced cell adhesion molecular relationship in HCC was identified including different molecules but same GO term and same molecule but different GO terms from the same activated* PTHLH* GO-molecular network of HCC compared with the corresponding activated GO-molecular network of no-tumor hepatitis/cirrhotic tissues, and constructed network by GRNInfer [11] and our articles [12–25] and illustrated by GVedit tool.

## 3. Results

Biological processes and occurrence numbers of the same activated high expression (fold change ≥2) *PTHLH *feedback-mediated cell adhesion GO network in HCC were identified and computed compared with the corresponding low expression activated GO network of no-tumor hepatitis/cirrhotic tissues (HBV or HCV infection), the different compared with the corresponding inhibited *PTHLH* feedback-mediated cell adhesion GO network of no-tumor hepatitis/cirrhotic tissues, and the same compared with the corresponding inhibited GO network of HCC, respectively.

The same biological processes of activated* PTHLH *feedback-mediated cell adhesion GO network in HCC included anaphase-promoting complex-dependent proteasomal ubiquitin-dependent protein catabolism, cell adhesion, cell differentiation, cell-cell signaling, endothelial cell migration, G-protein-coupled receptor protein signaling pathway, G-protein signaling, intracellular transport, metabolism, phosphoinositide-mediated signaling, positive regulation of transcription, protein amino acid phosphorylation, regulation of cyclin-dependent protein kinase activity, regulation of transcription, signal transduction, transcription, and transport compared with the corresponding activated GO network of no-tumor hepatitis/cirrhotic tissues. 

The different biological processes of activated* PTHLH *feedback-mediated cell adhesion GO network in HCC contained integrin-mediated signaling pathway, intracellular transport, microtubule cytoskeleton organization and biogenesis, regulation of cell growth, regulation of cyclin-dependent protein kinase activity compared with the corresponding inhibited GO network of no-tumor hepatitis/cirrhotic tissues.

The same biological processes of activated* PTHLH *feedback-mediated cell adhesion GO network in HCC included anaphase-promoting complex-dependent proteasomal ubiquitin-dependent protein catabolism, cell adhesion, cell differentiation, cell-cell signaling, DNA repair, G-protein-coupled receptor protein signaling pathway, integrin-mediated signaling pathway, metabolism, nucleotide and nucleic acid metabolism, oxidation reduction, phosphoinositide-mediated signaling, positive regulation of transcription, protein modification, proteolysis, regulation of cyclin-dependent protein kinase activity, regulation of transcription, signal transduction, and transcription, transport compared with the corresponding inhibited GO network of HCC, as shown in Table 1.

Activated *PTHLH* feedback-mediated cell adhesion molecular network and numbers in HCC were extracted and computed from the same activated* PTHLH* GO-molecular network of HCC compared with the corresponding activated GO-molecular network of no-tumor hepatitis/cirrhotic tissues. Our result showed that *PTHLH *feedback-mediated cell adhesion molecular network consisted of *BUB1B, GNG10, PTHR2, GNAZ, RFC4, UBE2C, NRXN3, BAP1, PVRL2, TROAP, VCAN, CCNA2, CDC6, CDKN2C,* and *ENAH *in HCC, as shown in Table 2. 

## 4. Discussion

 Our aim is to study novel high expression-activated* PTHLH *feedback-mediated cell adhesion mechanism in HCC. In this study, biological processes and occurrence numbers of the same activated high expression (fold change ≥2) *PTHLH *feedback-mediated cell adhesion GO network in HCC were identified and computed compared with the corresponding low expression activated GO network of no-tumor hepatitis/cirrhotic tissues (HBV or HCV infection), the different compared with the corresponding inhibited *PTHLH* feedback-mediated cell adhesion GO network of no-tumor hepatitis/cirrhotic tissues, and the same compared with the corresponding inhibited GO network of HCC, respectively (Table 1). 

Simultaneous occurrence of biological processes was identified between the same activated* PTHLH *feedback-mediated cell adhesion GO network of HCC (compared with the corresponding activated GO network of no-tumor hepatitis/cirrhotic tissues) and the different (compared with the corresponding inhibited *PTHLH *feedback-mediated cell adhesion GO network of no-tumor hepatitis/cirrhotic tissues), or the same (compared with the corresponding inhibited GO network of HCC) for putting forward hypothesis of activated* PTHLH *coupling feedback phosphoinositide to G-protein receptor signal-induced cell adhesion network, respectively.

Simultaneous occurrence of biological processes consisted of intracellular transport, regulation of cyclin-dependent protein kinase activity between the same activated* PTHLH *feedback-mediated cell adhesion GO network of HCC (compared with the corresponding activated GO network of no-tumor hepatitis/cirrhotic tissues) and the different (compared with the corresponding inhibited *PTHLH *feedback-mediated cell adhesion GO network of no-tumor hepatitis/cirrhotic tissues). 

Simultaneous occurrence of biological processes included anaphase-promoting complex-dependent proteasomal ubiquitin-dependent protein catabolism, cell adhesion, cell differentiation, cell-cell signaling, G-protein-coupled receptor protein signaling pathway, metabolism, phosphoinositide-mediated signaling, positive regulation of transcription, regulation of cyclin-dependent protein kinase activity, regulation of transcription, signal transduction, transcription, transport between the same activated* PTHLH *feedback-mediated cell adhesion GO network of HCC (compared with the corresponding activated GO network of no-tumor hepatitis/cirrhotic tissues), and the same (compared with the corresponding inhibited GO network of HCC).

The studies of phosphoinositide with adhesion are presented as follows. Phosphoinositide lipid phosphatase SHIP1 and PTEN coordinate to regulate cell migration and adhesion [26], TAPP2 links phosphoinositide 3-kinase signaling to B-cell adhesion through interaction with the cytoskeletal protein utrophin: expression of a novel cell adhesion-promoting complex in B-cell leukemia [27], neuregulin-1 regulates cell adhesion via an ErbB2/phosphoinositide-3 kinase/Akt-dependent pathway: potential implications for schizophrenia and cancer [28], stromal cell-derived factor-1alpha stimulates tyrosine phosphorylation of multiple focal adhesion proteins and induces migration of hematopoietic progenitor cells: roles of phosphoinositide-3 kinase and protein kinase C [29], and functional association of platelet endothelial cell adhesion molecule-1 and phosphoinositide 3-kinase in human neutrophils [30]. Therefore, we proposed activated* PTHLH *coupling feedback phosphoinositide to G-protein receptor signal-induced cell adhesion network in HCC.

**Figure 1:**
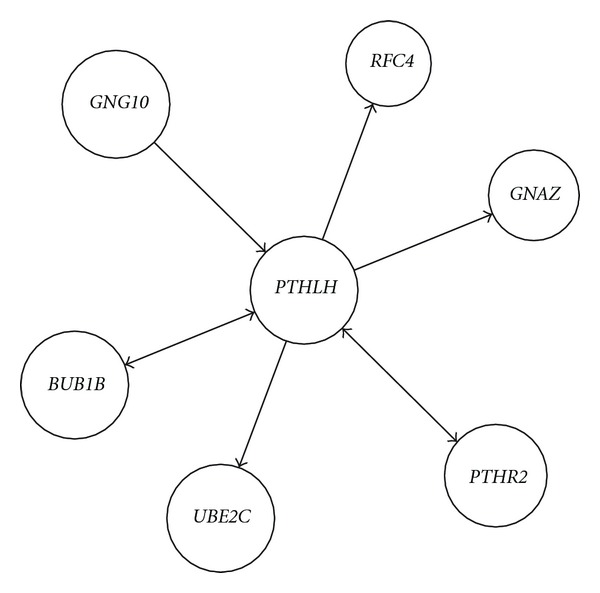
Activated* PTHLH *coupling feedback phosphoinositide to G-protein receptor signal network construction including different molecules but same GO term and same molecule but different GO terms in HCC from the same activated* PTHLH* GO-molecular network of HCC compared with the corresponding activated GO-molecular network of no-tumor hepatitis/cirrhotic tissues by GRNInfer and our programming.

**Figure 2:**
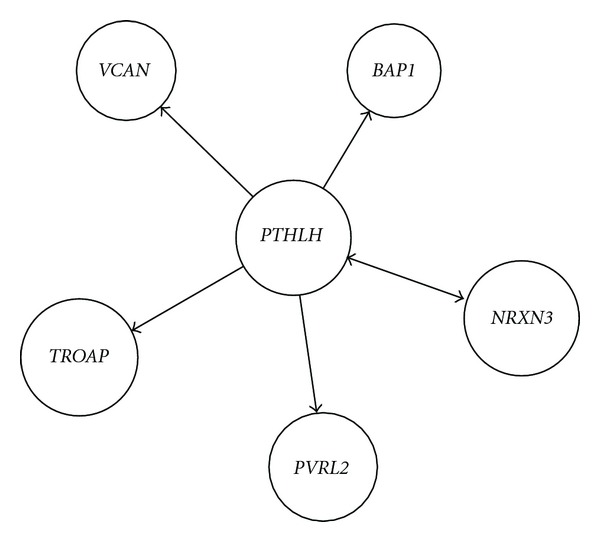
Activated* PTHLH *feedback cell adhesion network construction including different molecules but same GO term and same molecule but different GO terms in HCC from the same activated* PTHLH* GO-molecular network of HCC compared with the corresponding activated GO-molecular network of no-tumor hepatitis/cirrhotic tissues by GRNInfer and our programming.

Activated *PTHLH* feedback-mediated cell adhesion molecular network and numbers in HCC were extracted and computed from the same activated* PTHLH* GO-molecular network of HCC compared with the corresponding activated GO-molecular network of no-tumor hepatitis/cirrhotic tissues (Table 2)*. PTHLH* coupling feedback phosphoinositide to G-protein receptor signal-induced cell adhesion molecular relationship in HCC was identified including different molecules but same GO term and same molecule but different GO terms from the same activated* PTHLH* GO-molecular network of HCC compared with the corresponding activated GO-molecular network of no-tumor hepatitis/cirrhotic tissues. Activated* PTHLH *coupling feedback phosphoinositide to G-protein receptor signal network included *BUB1B, GNG10, PTHR2, GNAZ, PTHR2, BUB1B, RFC4,* and *UBE2C* and activated* PTHLH *feedback cell adhesion network *NRXN3, BAP1, NRXN3, PVRL2, TROAP,* and * VCAN *in HCC, as shown in Figures 1 and 2.

 In summary, studies were done on analysis of biological processes in the same high expression (fold change ≥2) activated* PTHLH *feedback-mediated cell adhesion GO network of HCC compared with the corresponding low expression activated GO network of no-tumor hepatitis/cirrhotic tissues (HBV or HCV infection). Activated* PTHLH *feedback-mediated cell adhesion network consisted of anaphase-promoting complex-dependent proteasomal ubiquitin-dependent protein catabolism, cell adhesion, cell differentiation, cell-cell signaling, G-protein-coupled receptor protein signaling pathway, intracellular transport, metabolism, phosphoinositide-mediated signaling, positive regulation of transcription, regulation of cyclin-dependent protein kinase activity, regulation of transcription, signal transduction, transcription, and transport in HCC. We proposed activated* PTHLH *coupling feedback phosphoinositide to G-protein receptor signal-induced cell adhesion network. Our hypothesis was verified by the different activated* PTHLH *feedback-mediated cell adhesion GO network of HCC compared with the corresponding inhibited GO network of no-tumor hepatitis/cirrhotic tissues, or the same compared with the corresponding inhibited GO network of HCC. Activated* PTHLH *coupling feedback phosphoinositide to G-protein receptor signal-induced cell adhesion network included *BUB1B, GNG10, PTHR2, GNAZ, RFC4, UBE2C, NRXN3, BAP1, PVRL2, TROAP,* and *VCAN *in HCC from GEO data set using gene regulatory network inference method and our programming.

## Figures and Tables

**Table 1:** (a) Biological processes and occurrence numbers of the same activated high expression (fold change ≥2) *PTHLH *feedback-mediated cell adhesion GO network in HCC compared with the corresponding low expression activated GO network of no-tumor hepatitis/cirrhotic tissues (HBV or HCV infection), (b) the different compared with the corresponding inhibited *PTHLH* feedback-mediated cell adhesion GO network of no-tumor hepatitis/cirrhotic tissues, and (c) the same compared with the corresponding inhibited GO network of HCC by our programming.

(a) Biological process and occurrence number of GO term	
Anaphase-promoting complex-dependent proteasomal ubiquitin-dependent protein catabolism	5
Cell adhesion	8
Cell differentiation	2
Cell-cell signaling	5
Endothelial cell migration	2
G-protein-coupled receptor protein signaling pathway	4
G-protein signaling	2
Intracellular transport	2
metabolism	4
Phosphoinositide-mediated signaling	4
Positive regulation of transcription	3
Protein amino acid phosphorylation	8
Regulation of cyclin-dependent protein kinase activity	8
Regulation of transcription	8
Signal transduction	10
Transcription	8
Transport	2

(b) Biological process and occurrence number of GO term	

Integrin-mediated signaling pathway	2
Intracellular transport	2
Microtubule cytoskeleton organization and biogenesis	2
Regulation of cell growth	2
Regulation of cyclin-dependent protein kinase activity	8

(c) Biological process and occurrence number of GO term	

Anaphase-promoting complex-dependent proteasomal ubiquitin-dependent protein catabolism	5
Cell adhesion	8
Cell differentiation	2
Cell-cell signaling	5
DNA repair	2
G-protein-coupled receptor protein signaling pathway	4
Integrin-mediated signaling pathway	2
Metabolism	4
Nucleotide and nucleic acid metabolism	2
Oxidation reduction	5
Phosphoinositide-mediated signaling	4
Positive regulation of transcription	3
Protein modification	2
Proteolysis	5
Regulation of cyclin-dependent protein kinase activity	8
Regulation of transcription	8
Signal transduction	10
Transcription	8
Transport	2

**Table 2:** Activated* PTHLH *feedback-mediated cell adhesion molecular network and numbers in HCC from the same activated* PTHLH* GO-molecular network of HCC compared with the corresponding activated GO-molecular network of no-tumor hepatitis/cirrhotic tissues by our programming.

Molecular name and number	
*BUB1B, GNG10, PTHR2, GNAZ, RFC4, UBE2C, NRXN3, BAP1, PVRL2, TROAP, VCAN, CCNA2, CDC6, CDKN2C, ENAH*	15
